# Study on the Influence of Surface Temperature Field of Aluminum Alloy Etched by Laser Water Jet Composite Machining

**DOI:** 10.3390/ma13143206

**Published:** 2020-07-18

**Authors:** Xuehui Chen, Xin Xu, Wei Liu, Lei Huang, Hao Li, Chao Wu, Weihao Mu, Xiang Li

**Affiliations:** 1 Key Laboratory of Intelligent Manufacturing of Construction Machinery, Anhui Jianzhu University, Hefei 230601, China; chenxuehui@ahjzu.edu.cn (X.C.); xuxin@ahjzu.edu.cn (X.X.); huangl75@ahjzu.edu.cn (L.H.); lihao402@aliyun.com (H.L.); cwhf2020@yeah.net (C.W.); muweihao@yeah.net (W.M.); 2 School of Mechanical Engineering, Hefei University of technology, Hefei 230009, China; 2019010015@mail.hfut.edu.cn

**Keywords:** water-jet-assisted laser processing, aluminum alloy, temperature field detection system, heat transfer mechanism

## Abstract

This paper studies the compound effect of liquid medium and laser on the workpiece and analyses the law of material surface temperature change during the processing. Taking 7075-T6 aluminum alloy as the research object, the surface temperature field of aluminum alloy processed using water-jet-assisted laser machining under different process parameters was simulated using finite element software. In addition, the temperature field of the material surface was detected in real-time using the self-built water-jet-assisted laser machining temperature field detection system, and the processing results were observed and verified using an optical microscope, scanning electron microscope, and energy spectrum analyzer. The results show that when the water jet inflow angle is 45°, the heat-affected area of the material surface is the smallest, and the cooling effect of the temperature field of the material surface is better. Considering the liquidus melting point of 7075 aluminum alloys, it is concluded that the processing effect is better when the water jet velocity is 14 m·s^−1^, the laser power is 100 W, and the laser scanning speed is 1.2 mm·s^−1^. At this time, the quality of the tank is relatively good, there are no cracks in the bottom of the tank, and there is less slag accumulation. Compared with anhydrous laser etching, water-jet-assisted laser etching can reduce the problems of micro-cracks, molten slag, and the formation of a recast layer in laser etching and improve the quality of the workpiece, and the composition of the bottom slag does not change. This study provides theoretical guidance and application support for the selection and optimization of process parameters for water-jet-assisted laser etching of aluminum alloy and further enriches the heat transfer mechanism of multi-field coupling in the process of water-jet-assisted laser machining.

## 1. Introduction

As a high-strength aluminum alloy, the 7075 aluminum alloy has excellent mechanical properties, such as good wear resistance, corrosion resistance, and oxidation resistance. This alloy is widely used in aerospace, mold processing, mechanical equipment [1,2,3], and other fields. Traditional mechanical processing inevitably has shortcomings, such as deformation and internal stress [4,5,6], but laser processing can solve these difficulties. However, due to the characteristics of high laser energy density, the heat-affected zone is larger, and even more, a serious ablation phenomenon occurs in the machining process [7,8,9]. Water-jet-assisted laser processing is a laser composite processing method developed in recent years. In the processing of high strength materials, water-jet-assisted laser processing can effectively reduce the heat-affected zone, reduce the temperature gradient, and wash away the molten slag [10] in time, to improve the processing quality.

At present, many researchers have done a lot of research on water-jet-assisted laser processing and made good progress. Kruusing et al. [11,12] produced a more detailed summary of water-jet-assisted laser processing technology and underwater laser processing technology based on previous research. Jyri et al. [13] carried out a series of experimental studies by using water-guided laser processing technology and obtained the influence of cutting angle on the surface quality of the workpiece. Tangwarodomnukun et al. [10,14] studied a water-jet-assisted micromachining technology, compared with anhydrous laser machining technology and laser composite machining technology, analyzed the effects of different process parameters on the heat-affected zone and machining quality, and improved the traditional water-jet-assisted laser machining, which is more beneficial to the removal of molten slag and further improves the surface quality. Chen et al. [15] used the orthogonal test method to perform low-pressure water-jet-assisted laser etching on alumina ceramic materials and studied laser parameters such as laser pulse width, laser repetition frequency and current, and water jet velocity on etching oxidation. The study of the influence of the surface quality of aluminum ceramics found that the use of low-pressure water-jet-assisted laser etching to process alumina ceramics can reduce the generation of micro-cracks and the accumulation of slag on the processed surface, yielding good processing quality. Mullick [16] established the analytical model of water-jet-assisted laser processing and found that 40% to 50% of the laser energy loss come from the vaporization of water. Zhu et al. [17] experimentally studied the effects of nanosecond laser and hybrid laser water-jet technology on the processing performance of germanium microgrooves and also analyzed and discussed the effects of laser pulse energy, pulse overlap, and focal plane position on the groove geometry and heat. The research results show that hybrid laser water jet technology can produce narrow and deep microgrooves in the smallest heat-affected zone. Eddie et al. [18] studied the effects of different jet velocity and nozzle diameter on the results of water-jet-assisted laser machining and optimized the jet velocities and nozzle diameter through experiments. Madhukar et al. [19] studied the effects of water-jet-assisted optical fiber laser processing and high-pressure gas-assisted optical fiber laser processing on the processing results of low carbon steel and titanium metal, respectively. It was found that due to the energy loss caused by the introduction of a water jet into the laser when the two methods were used to remove metal materials per unit volume, the laser energy of water-jet assisted optical fiber laser cutting is more than that of high-pressure gas-assisted optical fiber laser cutting. Wang et al. [20] proposed an improved water-jet-assisted laser machining for silicon nitride ceramic micromachining, in which the laser plays the role of softening material, and the water jet plays the role of cooling and scouring the laser softening material. The depth and surface micro-morphology of the processing groove using the response surface method and the effects of parameters such as laser pulse energy and water-jet pressure processing speed on the processing results have been studied. The experimental results show that the processing parameters have a direct influence on the groove depth and micro-morphology, in which the laser pulse energy and water-jet pressure play a major role in all the influencing factors. Chen et al. [21,22] used low-pressure water-jet-assisted laser etching of polysilicon materials and studied the changes of etching groove depth, volume, and laser energy utilization efficiency under multiple process parameters. The results show that low-pressure water-jet-assisted laser etching technology can improve the problems of traditional laser etching, such as micro-cracking, slagging, and recast layer formation, to improve the quality of machined parts.

The above scholars have mainly carried out experimental research on the influence of process parameters on machining quality, but there are few reports on the simulation and experimental research of the material surface temperature field in the water-jet-assisted machining process. In this paper, the surface temperature field of 7075 aluminum alloys processed using water-jet-assisted laser machining is simulated, and the related test platform is built. The change law of the surface temperature field of anhydrous and water-jet-assisted laser machining materials under different process parameters is studied.

## 2. Theoretical Analysis of Water Jet Action

Water-jet-assisted laser processing is the addition of a water jet to traditional laser processing, which can not only maintain the advantages of laser processing but also remove the slag and debris produced in laser processing by making use of the impact effect of the water jet, to a certain extent, improving the quality of material processing [22]. Due to the high temperature of the laser action area, the water jet impinging the laser action area will produce a vapor film between the processing areas, and the energy in the processing process will be lost through the convection heat transfer in the vapor film. Among them, the convective heat transfer of water on the material surface affects the laser energy utilization rate. Large convective heat transfer will bring some loss to the laser and the heat from the surface of the material. The calculation of convective heat transfer is to obtain the heat transfer coefficient between the water jet and the material surface and derive the heat transfer coefficient at different jet velocities, which satisfies the following equation [21,23,24]:

(1) h={0.715(vlu)1/2×Pr0.4×λ×l−1  0.15≪Pr≪30.715(vlu)1/2×Pr1/3×λ×l−1 Pr>3

Here, *h* is the surface heat transfer coefficient (W·m^−2^·K^−^^1^), *v* is the velocity of liquid along the surface of the material (m·s^−1^), *l* is the length of the plate in the direction of the flow velocity (m), *µ* is the dynamic viscosity coefficient of water (Ns·m^−2^), *Pr* is the Prandtl numbers of water and *λ* is the coefficient of thermal conductivity (W/m·K). Therefore, the surface heat transfer coefficient *h* is related to the velocity *v*, and the increase of *v*, *h* will continue to increase. An increase of the surface heat transfer coefficient leads to an increase of the energy loss in the laser processing area and a decrease of the temperature gradient in the processing area, which is helpful to reduce the thermal stress of the material and prevent serious ablation or cracks in the processing area.

**Figure 1:**
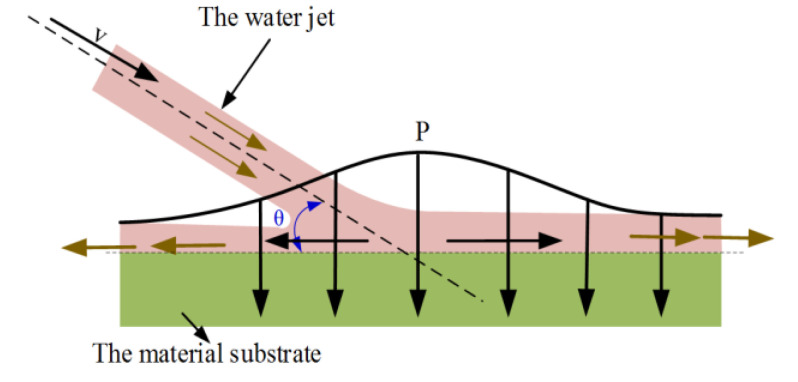
Schematic diagram of water jet impact.

In water-jet-assisted laser processing, the laser plays the role of heating and softening the material [21]. When the water-jet hits the processing area for cooling, its kinetic energy is bound to have a continuous impact on the processing area, and a schematic diagram of the impact material is shown in Figure 1. Due to the limited blowing capacity of the auxiliary gas, with the help of the impact of the water jet, the molten material produced by the laser, the molten slag attached to both sides of the tank, and the recast layer can be washed away from the tank body in time, and the processing quality of the tank body can be improved. Moreover, when the water jet hits the solid surface at high speed, the impact will produce water hammer pressure at the center of the jet impact, which plays an important role in material damage [25]. The formula of water hammer pressure is:

(2) $$P=\frac{E\left(v_{0}\times \sin\theta \right)}{\omega }$$

Here, *E* is the elastic modulus of the liquid, *ω* is the speed of sound in the jet, and *θ* is the angle of water jet inflow. It is assumed that the water jet has no loss in the air, that is, *v_0_* is the initial velocity of the water jet leaving the nozzle.

It can be seen from Equation (2) that with the continuous increase of jet velocity, the greater the water hammer pressure produced by the water jet impinging material and the larger the injection angle at the same injection velocity, the greater the water hammer pressure and the more obvious the impact effect. In summary, the increase of water jet velocity will lead to an increase of the impact force on the material surface and the heat transfer coefficient between the water jet and the material surface, and there is a positive correlation between the convective heat transfer coefficient and the flow velocity on the material surface. Therefore, the increase of water jet velocity will not only increase its impact on the laser processing process, but also increase the convective heat transfer and improve the cooling capacity of the material surface. For the same water jet velocity, the larger the incident angle is, the better the impact effect is, but the relationship between the water jet velocity along the material surface and the incident angle needs to be further studied below.

## 3. Simulation of the Temperature Field of 7075 Aluminum Alloy Surface by Water Jet Assisted Laser Etching

### 3.1. Water Jet Impact Simulation

**Figure 2:**
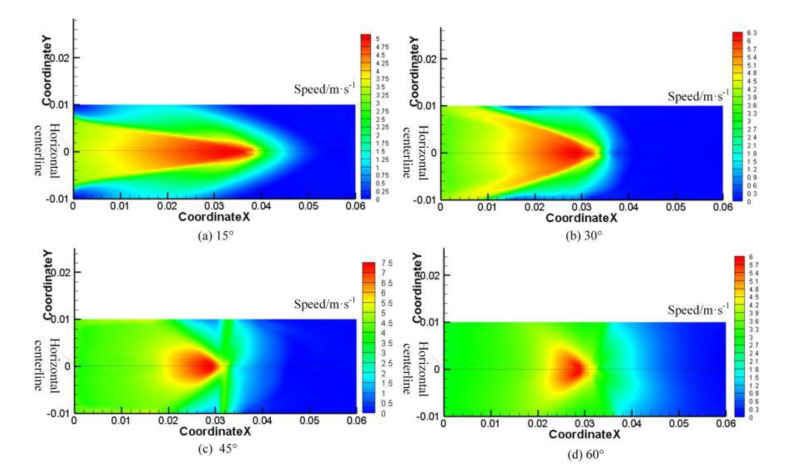
Velocity distribution of the water jet impacting the surface of the materials at different angles. (**a**) 15°; (**b**) 30°; (**c**) 45°; (**d**) 60°.

The research object of this numerical simulation is 7075-T6 aluminum alloy (length × width × height: 60 × 20 × 5 mm^3^), and the related basic parameters are shown in Table 1. A cylindrical nozzle with a cross section of 0.5 mm in diameter was also used. In the finite element software Fluent, the VOF model is used to simulate the velocity distribution of the water jet impinging on the surface of the material at the incident angles of 15°, 30°, 45°, and 60°, respectively. In the software, the water-jet inlet, the water jet nozzle wall, and the water-jet outlet are named respectively, and the outlet pressure is set to the standard atmospheric pressure, using the standard *k-ε* model. By the continuous modification of the model, we change the incident angle and keep the same conditions for the numerical simulation of water jet impact simulation. Figure 2 shows the velocity distribution of the water jet impacting the material surface at different angles.

**Figure 3:**
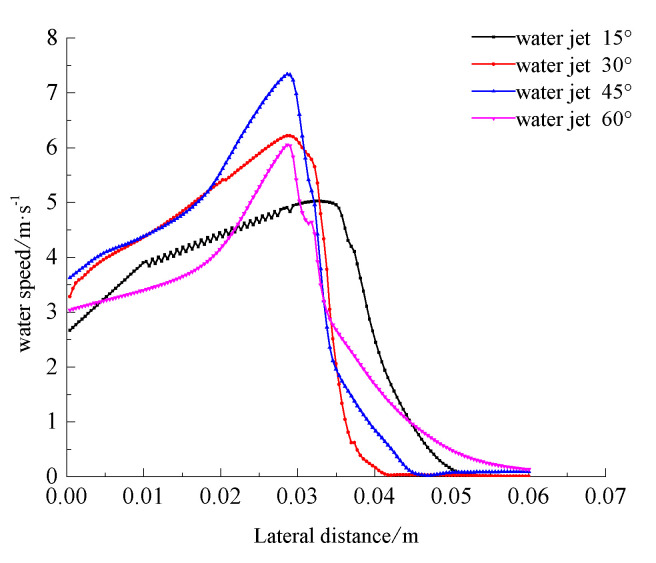
Curves of the relationship between the water velocity and the position of the horizontal centerline of the material surface at different angles.

Figure 2 shows that when the incident angle is increased from 15° to 45°, the larger the numerical distribution range of the water velocity on the surface of the aluminum alloy material is, the smaller the distribution range of the water jet velocity becomes. When the water jet angle is increased to 60°, the overall water velocity on the surface of the material is significantly smaller than when the water jet is at 45°. The analysis shows that as the angle of the water jet continues to increase, the force on the surface of the material will increase accordingly. This force will reduce the kinetic energy of the instantaneous water impacting the surface of the material. The reduction of the kinetic energy of the water will cause its speed to decrease during the forward flow, which will result in an overall reduction of the velocity of the water flowing at an angle of 60° [25]. We took the horizontal center line of the material surface and drew the curve of the relationship between the fluid velocity and the position of the material surface at different angles, as shown in Figure 3.

Figure 3 shows that the velocity distribution of the four angles of the water jet on the surface of the 7075 aluminum alloy material is such that the front of the impact gradually increases to a maximum value as the lateral center coordinate distance increases and then begins to decrease steadily. With different water jets, the maximum value corresponding to the angle varies. However, when the water jet angle is 15°, the water velocity on the surface of the centerline of the 7075 aluminum alloy material shows a clear upward and downward trend within the first half of the impact, which indicates that the water flow on the surface of the 7075 aluminum alloy material is more turbulent at this time. The other angles have no such obvious fluctuation trends. When the incident angle of the water jet is 30°, although its velocity is slightly larger than that of the 45° water jet at the front of the impact, the velocity value around the *x*-axis coordinate 0.03 of the core area of the impact when the water jet angle is 30° is significantly less than the 45° speed value. At the same time, the average values of the four water jet angles from 0 to 0.03 m are about 4.03 m·s^−1^ at 15°, about 4.88 m·s^−1^ at 30°, about 5.2 at 45°, and 4.04 m·s^−1^ at 60°. It can be seen that the water velocity on the surface of the 7075 aluminum alloy material is relatively large at the front of the impact (i.e., at a water jet incidence angle of 45° in the range of 0~0.03 m). When the incident angle of the water jet is 45°, the surface water velocity of the 7075 aluminum alloy material is larger than that at 30° and 60°, and the maximum value reaches 7.33 m·s^−1^. The convective heat transfer effect mainly depends on the velocity of the water jet. That is, the convective heat transfer effect is often better when the water jet velocity is higher, meaning that the cooling effect of the water-jet on the surface of the material is the best under these conditions.

### 3.2. Water Jet Assisted Laser Etching of the 7075 Aluminum Alloy Surface Temperature Field Analysis

To simulate the surface temperature field of materials using water-jet-assisted laser processing, the UDF function of Fluent software is needed to define the moving heat source. In the process of simulating water-jet-assisted laser etching, the VOF model is used to simulate water-jet-assisted laser machining. In water-jet-assisted laser etching, the higher the velocity of the water jet is, the higher the flow velocity of the water jet acting on the material surface, which enhances the cooling effect of the water jet on the material surface. However, in actual experiments, the water jet velocity will not increase continuously, and relevant studies [22,23] have shown that an excessively high water jet speed will reduce the laser processing effect. Therefore, the maximum initial velocity of the water jet set by the simulation is 18 m·s^−1^. At the same time, the control variable method is used to keep the other three sets of parameters unchanged. One set of parameters is changed to simulate the temperature field of the water-jet-assisted laser etching surface. The specific parameters are shown in Table 2.

To better express the change of material surface temperature field, the cloud diagram of the whole temperature distribution of the material surface, the magnification map of part of the region and the correlation curve diagram of the area of the surface temperature greater than 550 K and 750 K with the process parameters were drawn when the laser moved to the coordinate (0.02,0,0) under different process parameters. The area of the material surface greater than 550 K and 750 K is approximately considered as an ellipse, and its area is solved according to the calculation formula of the ellipse area:

(3) $$S=\frac{\pi \times A\times B}{4}$$

Here, *S* is the area of the ellipse, *A* is the long axis length of the ellipse, and *B* is the short axis length of the ellipse. The long axis and short axis of the ellipse are marked in the corresponding temperature field distribution cloud map.

**Figure 4:**
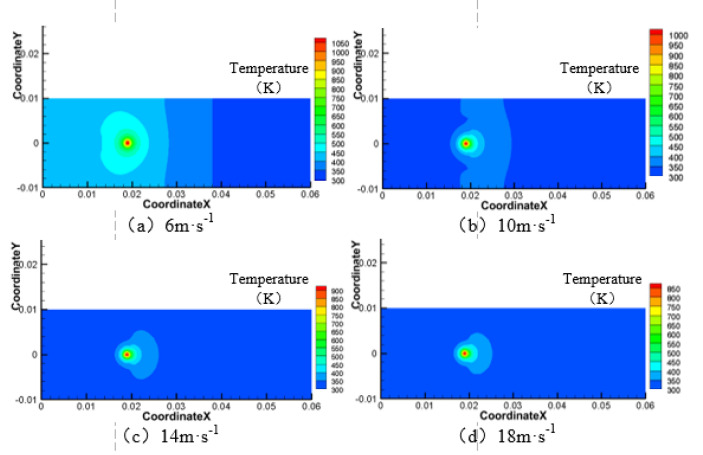
Surface temperature distribution of 7075 aluminum alloy at different water jet velocities. (**a**) 6 m·s^−1^; (**b**) 10 m·s^−1^; (**c**) 14 m·s^−1^; (**d**) 18 m·s^−1^.

**Figure 5:**
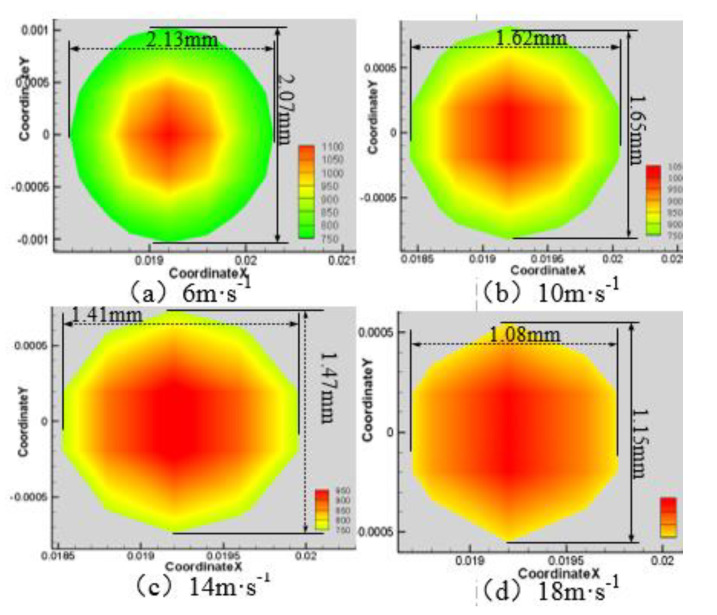
Material surface temperature distribution is more than 750 K at different water jet velocities. (**a**) 6 m·s^−1^; (**b**) 10 m·s^−1^; (**c**) 14 m·s^−1^; (**d**) 18 m·s^−1^.

**Figure 6:**
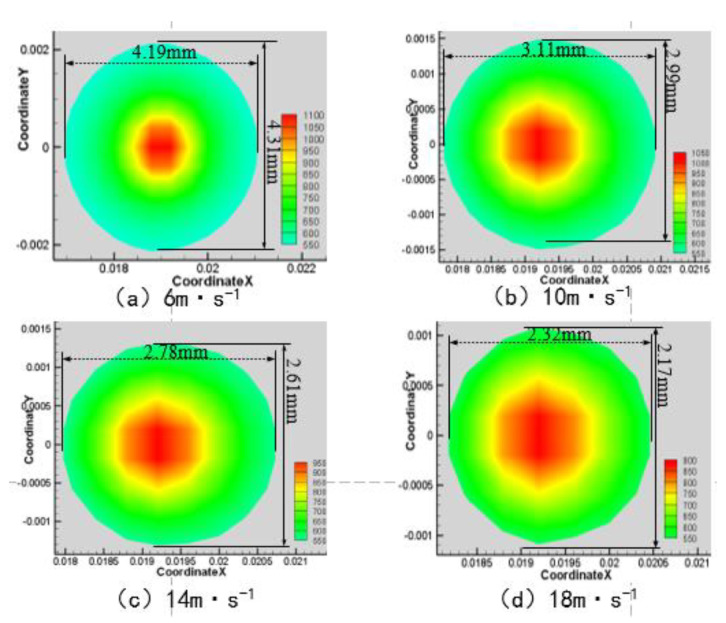
Material surface temperature distribution is more than 550 K at different water jet velocities. (**a**) 6 m·s^−1^; (**b**) 10 m·s^−1^; (**c**) 14 m·s^−1^; (**d**) 18 m·s^−1^.

**Figure 7:**
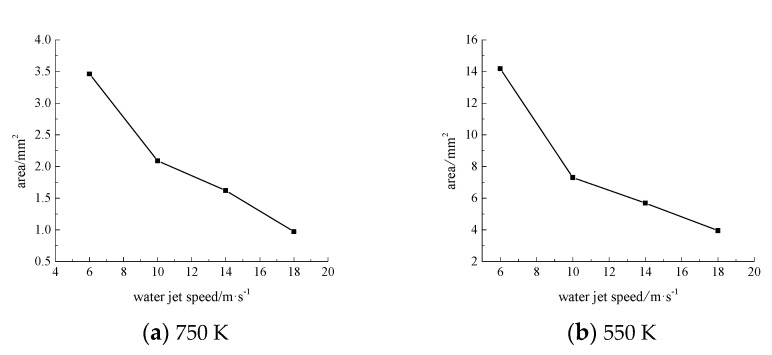
The surface area of the 7075 aluminum alloy with different water jet velocities is more than an area of 750 K (**a**) and 550 K (**b**).

When the laser power is 200 W, the angle of the water jet is 45°, and the laser scanning velocity is 0.8 mm·s^−1^. The area of the material surface temperature above 750 K and 550 K is shown in Figure 4, Figure 5, Figure 6 and Figure 7 when the water jet velocity is 6, 10, 14, and 18 m·s^−1^, respectively.

As can be seen from Figure 4, Figure 5, Figure 6 and Figure 7, when the laser power is 200 W, the angle of the water jet is 45°, and the laser scanning velocity 0.8 mm·s^−1^ is constant, the area of the material surface larger than 550 K and 750 K decreases with the increase of the water jet velocity. Considering the increasing velocity of the water jet, the convective heat transfer between the material surface and the water jet is gradually enhanced, which leads to a decrease of the maximum temperature and the overall temperature of the material surface. Therefore, with the continuous increase of the water jet velocity, the heat-affected zone of the machined surface can be effectively reduced.

**Figure 8:**
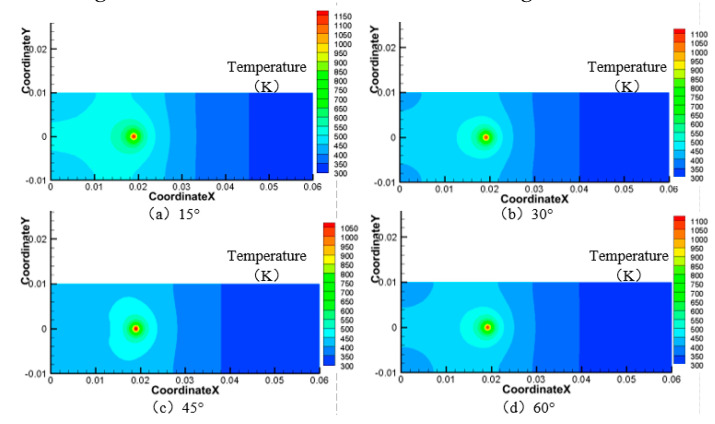
Surface temperature distribution of 7075 aluminum alloy at different water jet incident angles. (**a**) 15°; (**b**) 30°; (**c**) 45°; (**d**) 60°.

**Figure 9:**
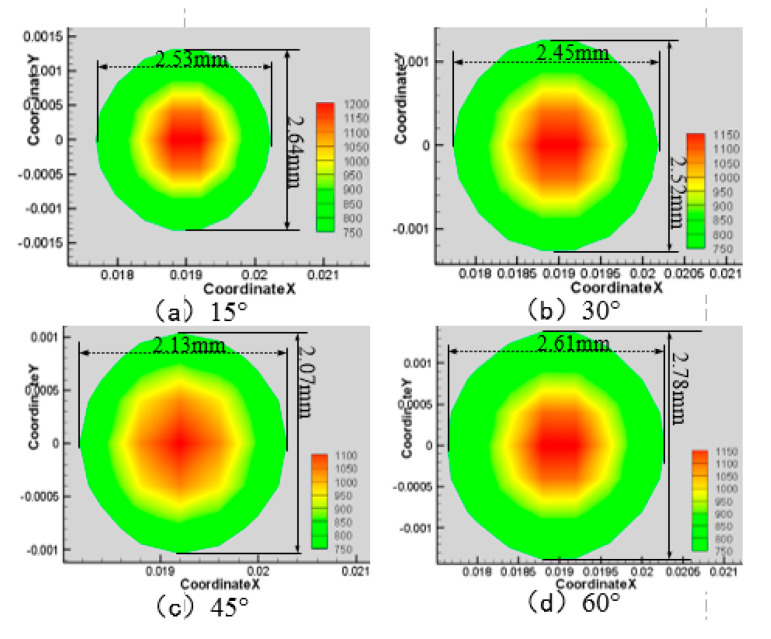
Material surface temperature distribution is more than 750 K at different water jet incident angles. (**a**) 15°; (**b**) 30°; (**c**) 45°; (**d**) 60°.

**Figure 10:**
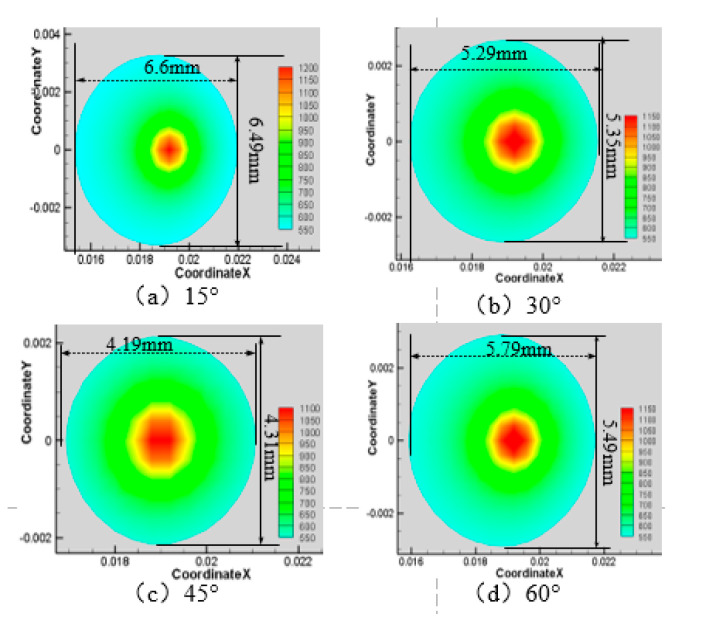
Material surface temperature distribution is more than 550 K at different water jet incident angles. (**a**) 15°; (**b**) 30°; (**c**) 45°; (**d**) 60°.

**Figure 11:**
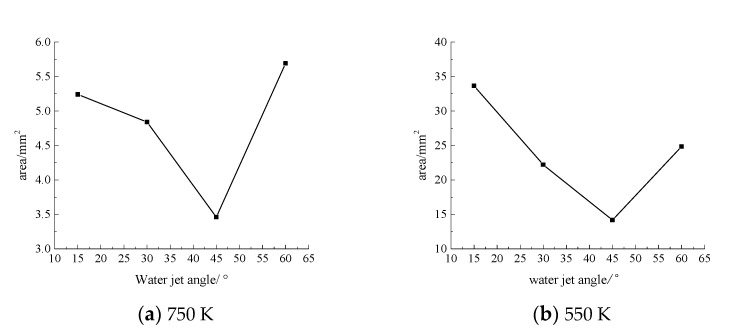
The surface area of the 7075 aluminum alloy with different water jet angles is more than the area of 750 K (**a**) and 550 K (**b**).

Next, the laser power is 200 W, the water jet velocity is 6 m·s^−1^, and the laser scanning speed is 0.8 mm·s^−1^, and when the water jet injection angle is 15°, 30°, 45°, and 60° respectively, the surface area of the material is larger than 750 K and 550 K, as shown in Figure 8, Figure 9, Figure 10 and Figure 11.

As can be seen from Figure 8, Figure 9, Figure 10 and Figure 11, when the water jet velocity is 6 m·s^−1^, the laser power is 200 W and the laser scanning speed 0.8 mm·s^−1^ is constant, the overall temperature of the material surface decreases with the increase of the incident angle from 15° to 45°, but when the incident angle increases to 60°, the overall temperature of the material surface begins to increase, which decreases at first and then increases. Considering that when the water jet inflow angle is 45°, the water velocity impacting on the material surface is relatively large, and the effect of convective heat transfer on the material surface is relatively large. At this time, the cooling effect of the material surface is the best. Therefore, when the incident angle is 45°, the overall temperature of the machined surface of the material is the minimum.

**Figure 12:**
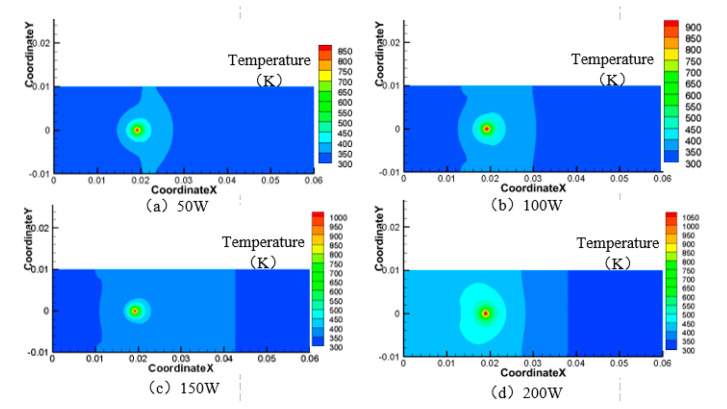
Surface temperature distribution of 7075 aluminum alloy at different laser power. (**a**) 50 W; (**b**) 100 W; (**c**) 150 W; (**d**) 200 W.

**Figure 13:**
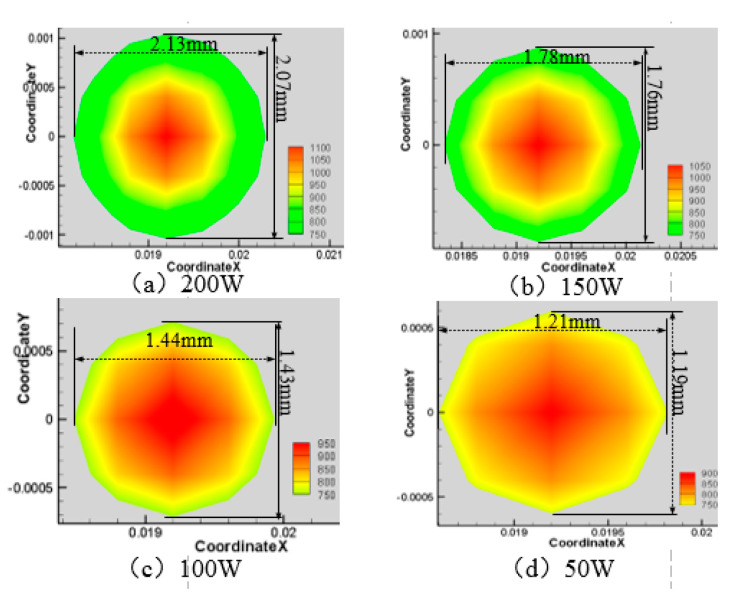
Material surface temperature distribution is more than 750 K at different laser power. (**a**) 200 W; (**b**) 150 W; (**c**) 100 W; (**d**) 50 W.

**Figure 14:**
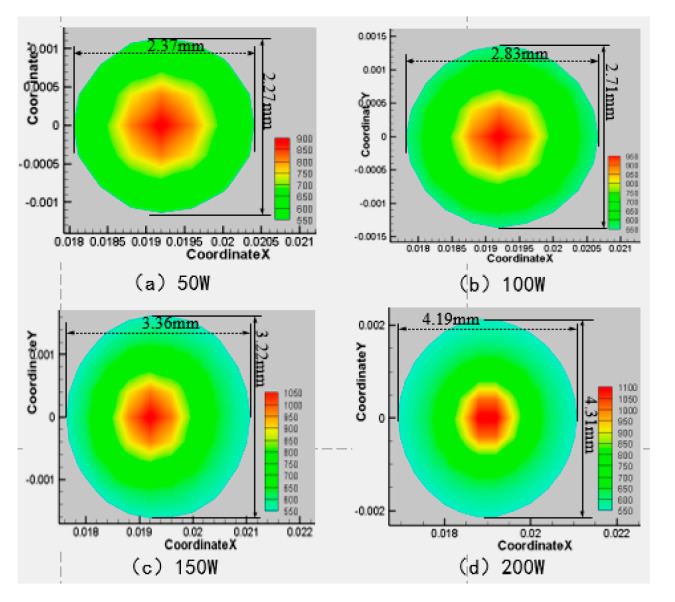
Material surface temperature distribution is more than 550 K at different laser power. (**a**) 50 W; (**b**) 100 W; (**c**) 150 W; (**d**) 200 W.

**Figure 15:**
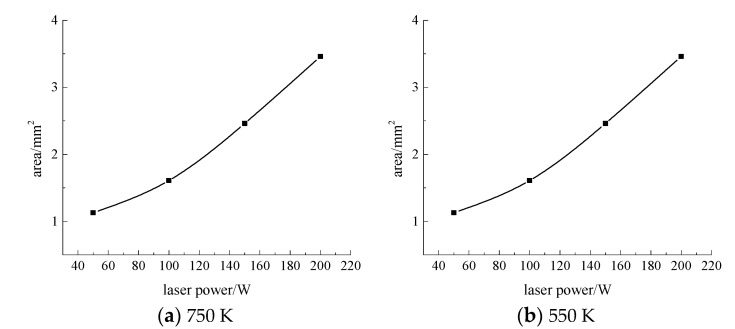
The surface area of the 7075 aluminum alloy with different laser powers is more than the area of 750 K (**a**) and 550 K (**b**).

Next, the velocity of the water-jet is 6 m·s^−1^, the angle of the water-jet is 45°, and the laser scanning speed is 0.8 mm·s^−1^, and the laser power is set at 50 W, 100 W, 150 W, and 200 W respectively. The area of the material surface greater than 750 K and 550 K is shown in Figure 12, Figure 13, Figure 14 and Figure 15.

As can be seen from Figure 12, Figure 13, Figure 14 and Figure 15, when the laser power increases from 50 W to 200 W, the range of surface temperature higher than 550 K and 750 K increases gradually. Considering that the cooling ability of the water jet on the material surface remains unchanged, the increase of the laser power leads to an increase of the total energy acting on the material surface, which leads to a continuous increase of the temperature of the material surface.

**Figure 16:**
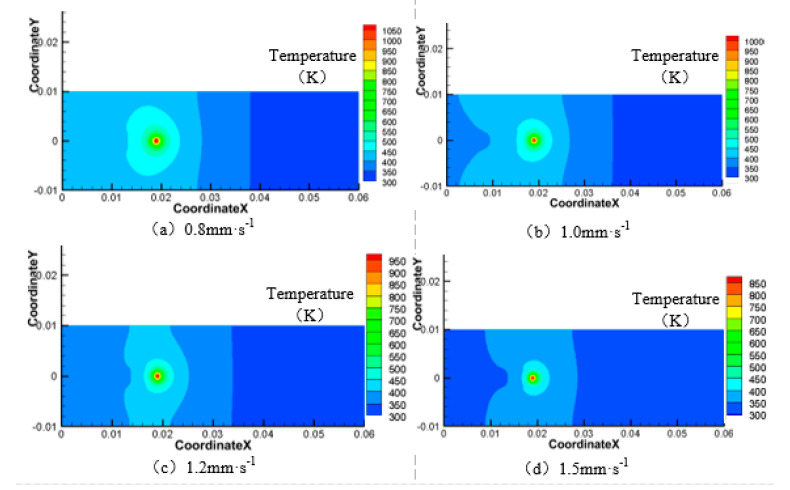
Surface temperature distribution of 7075 aluminum alloy at different laser scanning speeds. (**a**) 0.8 mm·s^−1^; (**b**) 1.0 mm·s^−1^; (**c**) 1.2 mm·s^−1^; (**d**) 1.5 mm·s^−1^.

**Figure 17:**
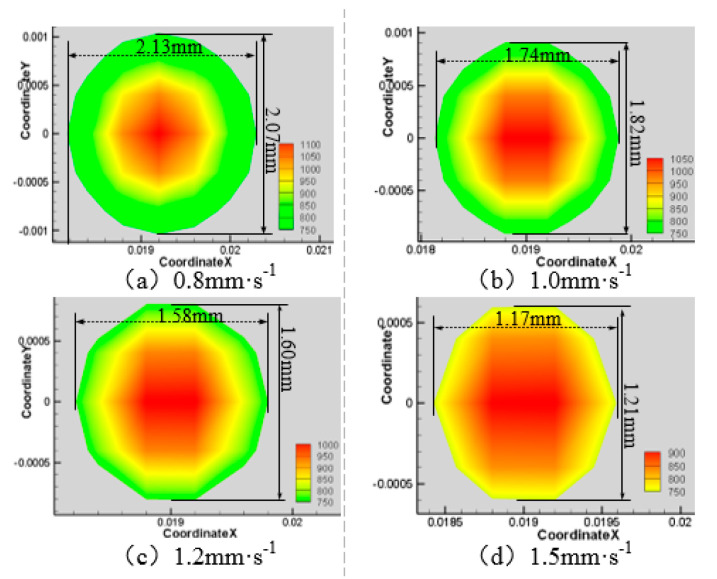
Material surface temperature distribution is more than 750 K at different laser scanning speeds. (**a**) 0.8 mm·s^−1^; (**b**) 1.0 mm·s^−1^; (**c**) 1.2 mm·s^−1^; (**d**) 1.5 mm·s^−1^.

**Figure 18:**
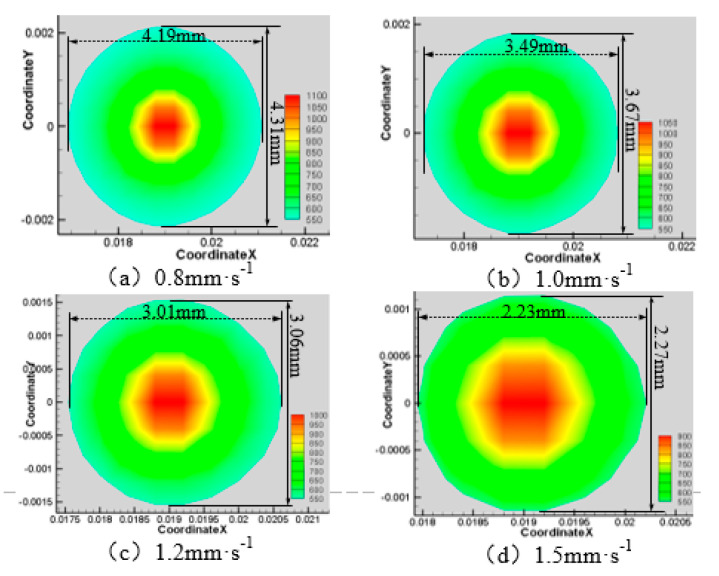
Material surface temperature distribution is more than 550 K at different laser scanning speeds. (**a**) 0.8 mm·s^−1^; (**b**) 1.0 mm·s^−1^; (**c**) 1.2 mm·s^−1^; (**d**) 1.5 mm·s^−1^.

**Figure 19:**
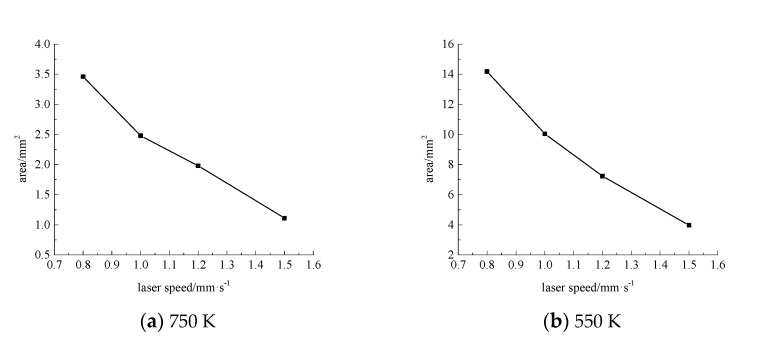
The surface area of the 7075 aluminum alloy with different laser scanning speeds is more than the area of 750 K (**a**) and 550 K (**b**).

Next, the laser power is 200 W, the water jet incident angle is 45°and the water jet velocity is 6 m·s^−1^, and the laser scanning speeds is 0.8 mm·s^−1^, 1.0 mm·s^−1^, 1.2 mm·s^−1^, and 1.5 mm·s^−1^, respectively. The area of the material surface greater than 750 K and 550 K is shown in Figure 16, Figure 17, Figure 18 and Figure 19.

As can be seen from Figure 16, Figure 17, Figure 18 and Figure 19, with the increase of laser scanning speed, the range of material surface temperature more than 550 K and 750 K decreases, which indicates that the material surface temperature decreases with the increase of laser scanning speed. This is because when the scanning speed increases, the time for the laser to move to the same position decreases, and because the laser power is constant, the total energy input by the laser will be reduced. Considering that the cooling effect of the water jet on the material surface remains unchanged, with the increase of the laser scanning speed, the material surface temperature will decrease and the heat-affected zone will decrease accordingly. To sum up, when the water jet velocity is 18 mm·s^−1,^ the incident angle is 45°, the laser scanning speed is 1.5 mm·s^−1^, and the laser power is 50 W, the range of the material surface above 550 K and 750 K is the smallest and the overall temperature is the lowest. However, considering that the liquidus melting point of 7075 aluminum alloy is 908 K, the surface temperature of the material processing area has not reached the liquidus melting point of the material, so the groove can not be formed on the material surface. Therefore, when the water jet velocity is 14 m·s^−1^, the incident angle is 45°, the scanning speed is 1.2 mm·s^−1^, and the laser power is 100 W, the heat-affected zone of the material surface is relatively minimal and the processing quality is the best.

## 4. Experimental Detection and Processing Results

### 4.1. Experimental Processing System

**Figure 20:**
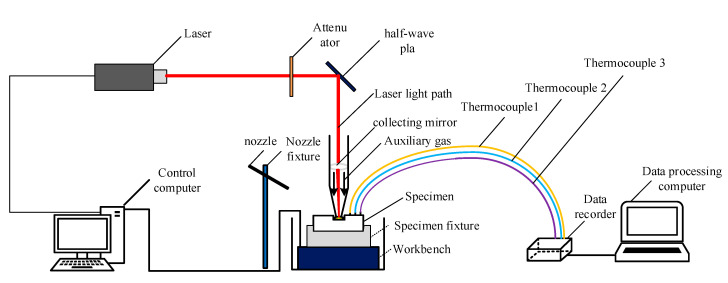
Schematic diagram of experimental processing system.

The experimental processing system mainly includes the Nd: YAG laser, a fixture, a protective device, a working table, water jet equipment, and temperature field detection equipment. A structural schematic diagram and partial physical diagram are shown in Figure 20.

**Figure 21:**
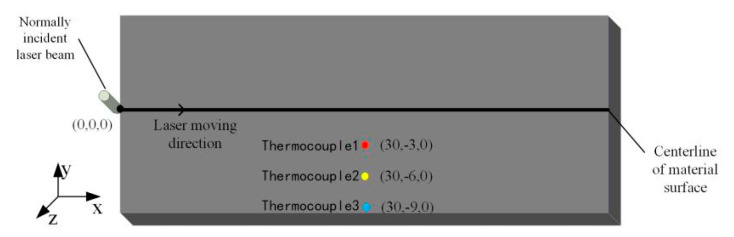
Schematic diagram of the specific location of the thermocouple welding.

This test used a TY-LFM-500 multi-function laser processing machine (Wuhan Tianzhiyi Technology Co., LTD, Wuhan, China). The laser was etched perpendicular to the surface of the test piece, the water jet was held in place by the fixture, and the angle was adjusted using the angle adjuster provided with the fixture. The three thermocouples were welded at the specified position shown in Figure 21, this work was specified by the laser control computer. The stage drives the test piece fixed on the working platform to carry out the specified movement. Because the water jet is faster in the actual processing process and the pressure in the water flow is relatively large, the water is difficult to measure with the traditional speed sensor, so the formulas were combined in this experiment:

(4) $$v_{0}=1.3\times \frac{V_{w}}{d^{2}\times t}$$

Here, *v*_0_ is the injection rate of the water jet, *V_w_* is the volume of water flowing out of the nozzle, *d* is the diameter of the nozzlet, and *t* is the measuring time. The pressure adjustment knob of the water pump can change the injection pressure of the nozzle outlet, as well as the size and range of the flow rate.

### 4.2. Water Jet Assisted Laser Etching of the 7075 Aluminum Alloy Surface Temperature Field Result Analysis

**Figure 22:**
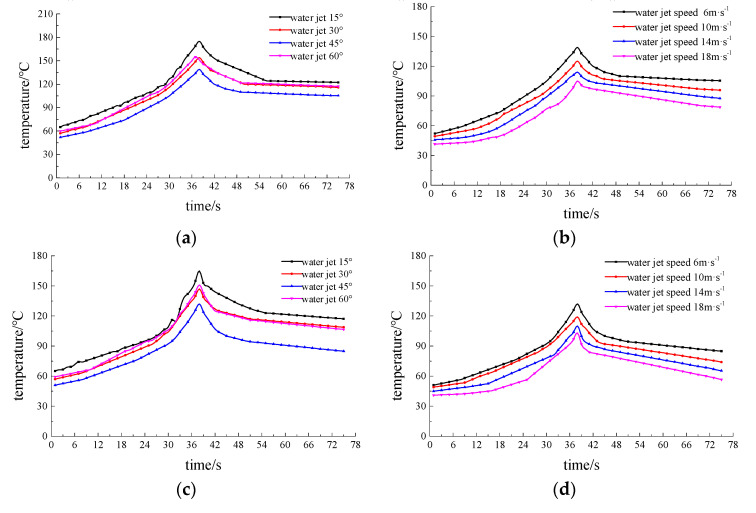

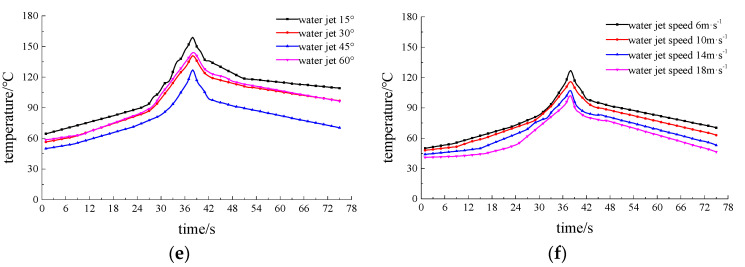
Measured values of the temperatures of three thermocouples in the composite processing of different water jet parameters. (**a**) Measured value of thermocouple 1 when the water jet angle is different. (**b**) Measured value of thermocouple 1 when the water jet speed is different. (**c**) Measured value of thermocouple 2 when the water jet angle is different. (**d**) Measured value of thermocouple 2 when the water jet speed is different. (**e**) Measured value of thermocouple 3 when the water jet angle is different. (**f**) Measured value of thermocouple 3 when the water jet velocity is different.

**Figure 23:**
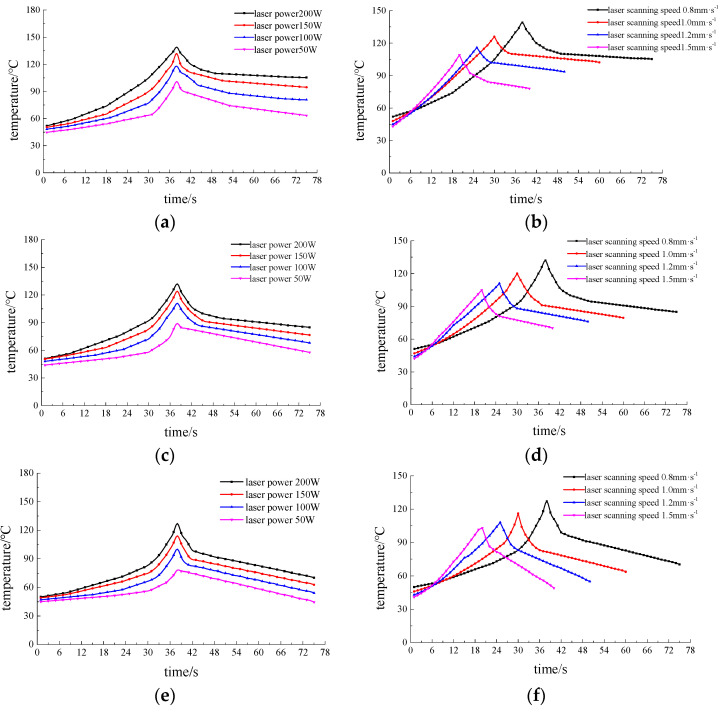
Measured values of the temperatures of the three thermocouples during the composite processing of the different laser parameters. (**a**) Measured values of thermocouple 1 with different laser powers. (**b**) Measured values of thermocouple 1 with different laser scanning speeds. (**c**) Measured values of thermocouple 2 with different laser powers. (**d**) Measured values of thermocouple 2 with different laser scanning speeds. (**e**) Measured values of thermocouple 3 with different laser powers. (**f**) Measured values of thermocouple 3 with different laser scanning speeds.

Figure 22 and Figure 23 show the temperature values measured by three thermocouples during water-jet-assisted laser machining with different process parameters. Among them, Figure 22a,c,e show the temperature measurements of thermocouples with laser scanning speed 0.8 mm·s^−1^, laser power 200 W, and water jet velocity 6 m·s^−1^ at different water jet angles. Figure 22b,d,f are laser scanning speed 0.8 mm·s^−1^, laser power 200 W, incident angle 45°, and thermocouple temperature measurements at different water jet velocities. Figure 23a,c,e are laser scanning velocity 0.8 mm·s^−1^, water jet velocity 6 m·s^−1^, incident angle 45°, and thermocouple temperature measurements at different laser powers. Figure 23b,d,f are laser power 200 W, water jet velocity 6 m·s^−1^, incident angle 45°, and temperature measurement of the thermocouple at different scanning speeds.

Figure 22a,c,e shows that the overall temperature of the three temperature measurement points decreases at first and then increases with the increase of water injection angle, it is relatively lowest when the injection angle is 45°, and the temperature values are almost the same when the water injection angle is 30° and 60°. At the same time, when the incident angle of the water jet is 15°, the temperature of the water jet shows an oscillating increase during part of the temperature rising stage from 0~38 s, while the other angles have no such trends. The cooling effect of the water jet on the material surface mainly depends on the convective heat transfer between the water and the material surface, and the effect of the convective heat transfer is mainly determined by the flow velocity distribution on the material surface. From the above simulation analysis, when the incident angle of the water jet is 15°, the distribution of the water velocity acting on the surface of the material is less uniform, resulting in a cooling effect in unit intervals. When the incident angle is 45°, the cooling effect on the material surface is the best, so the temperature of the three measuring points is the lowest. When the incident angle is 30° and 60°, the cooling effect on the material surface is similar.

It can be seen from Figure 22b,d,f that as the water jet velocity continues to increase, the overall temperature of the three temperature measurement points gradually decreases, and the temperature increase rate gradually decreases during the temperature rise at 0~38 s. This is because the convective heat transfer effect on the material surface is gradually strengthened with the increase in the water jet velocity, resulting in a continuous decrease in the overall temperature. At the same time, the cooling effect of the water jet on the 7075 aluminum alloy surface becomes enhanced by the improvement in convective heat transfer. At this time, the temperature measurement point temperature is relatively reduced by the increased cooling capacity of the water jet. When the velocity of the water jet increases, the velocity of the water behind the impact also increases accordingly. The trend of the temperature gradually decreasing at a later time becomes more obvious as the velocity of the water jet increases.

It can be seen from Figure 23a,c,e that as the laser power continues to increase, the maximum temperature and temperature increase rate measured by the three thermocouples during the temperature rise period of 0~38 s follows. At the same time, as the laser power decreases, the three thermocouples all show a downward trend in the later stages of the slow temperature change; the decrease rate of the measured value of the thermocouple temperature corresponding to a smaller power is greater than that at a higher power. This is because when the laser power is weakened, the energy that acts on the material will decrease accordingly. Thus, the surface temperature of the material will decrease, and the cooling capacity of the water jet will not change, making the maximum temperature of the material surface decrease accordingly. In Figure 23a,c,e, we can see that the three thermocouples slowly decrease in temperature at the same power temperature as thermocouple 1 (<thermocouple 2 <thermocouple 3). This is because thermocouple 1 is relative to thermocouple 2, which is closer to the straight-line distance of the real-time position of the laser spot. Under the same processing conditions and laser parameters, the energy delivered by the laser to thermocouple 1 per unit of time is relatively high, causing its temperature to be relatively high, which in turn leads to the measured temperature drop rate of the low-power thermocouple becoming greater than that of the larger power. The maximum temperature and temperature trend of the measured temperature of the lower power during processing show that the cooling effect of the water jet on the surface of the material gradually improves. The cooling effect of the 7075 aluminum alloy surface when the jet participates in the laser etching process gradually improves as the laser power decreases. It can be seen from Figure 23b,d,f that as the laser scanning speed continues to increase, the maximum temperature of the three temperature measurement points continues to decrease. Moreover, the range of temperature change increases continuously during the temperature decrease stage, which shows that with a continuous increase in the laser scanning speed, the cooling effect of the water jet on the material surface will be continuously improved.

### 4.3. Processing Quality and Energy Spectrum Analysis

**Figure 24:**
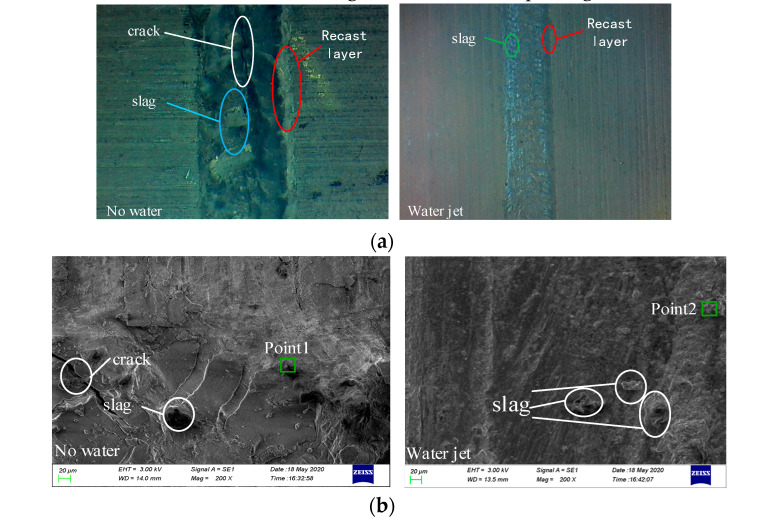
Non-water and water jet assisted laser processing tanks at different magnifications. (**a**) Drawing of the auxiliary laser processing tank with and without a water jet (×20). (**b**) Partial grooves of laser processing assisted with and without water jet (×200).

In Figure 24a,b, the laser power is 100 W, there is no water, the scanning speed is 1.2 mm·s^−1^, and the water jet participates in the laser etching processing. Further, the water jet incident angle is 45°, and the water jet speed is 14 m·s^−1^. The groove figure is formed by processing under these parameters. Figure 24a was taken under 20 times magnification, and Figure 24b presents a partial groove bottom view obtained under a scanning electron microscope magnification of 200 times.

It can be seen from Figure 24a that the water-assisted laser etching process has a wider groove width than the water-jet-assisted laser etching process, and more recast layers are generated on the side edges of the groove body; at the same time, the groove that the slag generated at the bottom is larger than that under the water jet assisted laser etching process, and the slag is mostly accumulated in blocks. Moreover, there are cracks at the bottom of the groove. At the same time, it can be seen from Figure 24a that the introduction of a water-jet in the laser etching process not only reduces the recast layer on the side edges of the tank body but also generates slag particles at the bottom of the tank; the results of water-assisted laser etching are relatively smaller. Comparing Figure 24a,b, it can be seen that the water-jet-assisted laser etching process significantly improved the processing quality compared to the anhydrous laser etching process. According to the analysis, during the anhydrous laser etching process, due to the high laser energy density and extreme cold and heat, a part of the melt is generated during the etching process, while the auxiliary gas has a limited blow-off capability and cannot be blown off in time. Part of the melt accumulates at the bottom of the tank to form slag, and the slag accumulated on the bottom of the tank further transfers heat to the inside of the material, making the internal thermal stress of the material too large and forcing the material to crack. At the same time, parts of the melt attach to the sides of the tank body to form a recast layer via the blowing effect of the auxiliary gas.

Figure 24a,b show that when a water jet was used to assist the laser etching, the instant cooling effect of the water jet strongly reduced the thermal stress produced during the process to avoid cracks at the bottom of the groove. The self-impact effect washed away the melt produced during the process over time to reduce the generation of slag and the recast layer. However, because the sample is a metal material, its thermal conductivity is high. Although the water jet can flush out the molten material produced in the production process, the water jet’s ability to remove the relevant material is reduced due to the existence of the tank. Thus, a small portion of the melt still accumulates on the bottom and wall of the tank to form a slag and recast layer. At the same time, due to the instant cooling effect of the water jet, the area of the sample surface above its melting point during laser etching is reduced—that is, the groove width formed by the processing becomes relatively narrow.

In order to further analyze the processing mechanisms under conditions with and without water jet assistance, the slag formed by the two processes was taken for EDS analysis. The specific measurement positions are shown as points 1 and 2 in Figure 24b.

**Figure 25:**
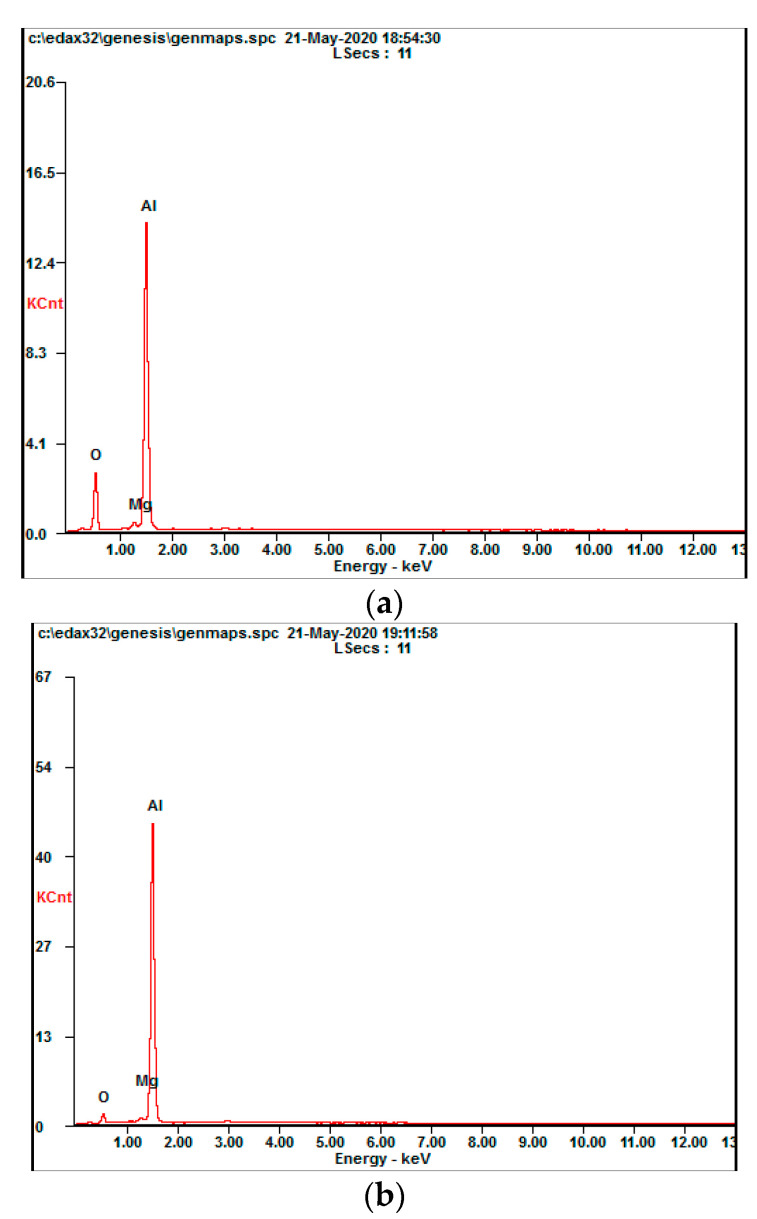
Scanning analysis of the EDS point of slag at the bottom of the tank obtained by laser processing the 7075 aluminum alloy with and without a water jet. (**a**) No water; (**b**) water jet.

It can be seen from Figure 25 and Table 3 that when the water-jet participates in the laser etching process, the main composition of the slag formed and deposited at the bottom of the groove does not change (the main zinc element in the 7075 aluminum alloy is present because the processing temperature is too high for the zinc to reach its own boiling point and evaporate). However, the proportion of its oxygen content increased significantly. At the same time, its aluminum content decreased, while its magnesium content remained almost unchanged. According to the analysis, when aluminum alloy materials are processed using anhydrous laser etching, the aluminum alloy materials are likely to form an aluminum oxide film in the air environment to cover the surface of the material to prevent it from continuing to undergo oxidation reactions inside the material. Magnesium is present, but the main component is still aluminum. The introduction of water jets causes part of the water jets to form water vapor due to the excessively high temperature in the processing area during laser processing. Related studies [26,27] have shown that magnesium further promotes aluminum and water vapor to undergo t related reaction processes.

The final product is Al_2_O_3_, Al_2.667_O_4_. Thus, when the water jet is added to the laser etching process, the oxygen element in the generated slag will increase, and the aluminum element content will decrease.

## 5. Conclusions

In this paper, aiming at water-jet-assisted laser etching of 7075 aluminum alloys, the surface temperature field of water-jet-assisted laser machining of aluminum alloy with different process parameters was simulated using finite element software, and verified using a self-built experimental device for temperature field detection. Finally, the processing results were compared and analyzed through an optical microscope, scanning electron microscope, and energy spectrum analyzer. The findings are summarized as follows:

(1) When the incident angle of the water jet is 45°, the speed of the water jet is 14 m·s^−1^, the laser power is 100 W, and the laser scanning speed is 1.2 mm·s^−1^, the quality of the processing groove is relatively good; there are no cracks at the bottom of the tank, and there is less slag accumulation.
(2) In the process of water-jet-assisted laser machining, the temperature of the middle monitoring point on the material surface increases at first and then declines with the laser processing time. The highest temperature is located in the intermediate time of laser scanning, and the maximum temperature increases with the increase of laser power and decreases with the increase of laser scanning speed. The velocity and incident angle of the water jet plays an important role in the cooling of the material surface. With the increase of the water jet velocity, the cooling effect on the material surface increases gradually, and with the increase of the water jet angle, the cooling effect on the material surface increases at first and then decreases. When the incident angle is 45°, the cooling effect on the temperature field of the material surface is better.
(3) In anhydrous laser machining, there are more recasting layers on both sides of the groove, and at the same time, more slag is produced at the bottom of the groove than in the water-jet-assisted laser machining, and there are cracks at the bottom of the groove. Water-jet-assisted laser machining greatly reduces the recasting layer on both sides of the groove, and the slag particles produced at the bottom of the groove are smaller than those of anhydrous assisted laser etching, and the width and depth of the groove are reduced. The quality of water-jet-assisted laser etching is better than that of anhydrous-assisted laser etching.
(4) In the process of water-jet-assisted laser machining, the main composition of the slag deposited at the bottom of the tank has does not changed. Due to the continuous erosion of water, the aluminum alloy material reacts with water at a high temperature, the content of the oxygen element increases significantly, the content of the aluminum element decreases relatively, while the content of the magnesium element remains almost unchanged.

The research results of this paper are helpful to grasp the dynamic distribution of the surface temperature field of the liquid medium and the law of heating and cooling in the processing area and provide an effective method for understanding the temperature field of liquid-assisted laser composite machining. At the same time, they also provide a reference for the further optimization of laser composite processing technology in the future.

## Figures and Tables

**Table 1:** 7075-T6 aluminum alloy material physical properties.

Density (g·cm^−^^3^)	2.8
Solidus melting point (°C) Liquidus melting point (°C)	477 635
Boiling point (°C)	2065
Antioxidant	good
Poisson’s ratio	0.33

**Table 2:** Control variable simulation parameter level table.

Serial Number	Water Speed (m∙s^−1^)	Impact Angle (°)	Laser Power (W)	Laser Scanning Speed (mm∙s^−1^)
1	6/10/14/18	45°	200	0.8
2	6	15°/30°/45°/60°	200	0.8
3	6	45°	50/100/150/200	0.8/1/1.0/1.5
4	6	45°	200	0.8

**Table 3:** Scanning analysis of the point 1 and 2 elements in Figure 14b.

Position	Element	at.%	wt.%	Position	Element	at.%	wt.%
1	O	17.74	11.36	2	O	48.69	36.07
	Mg	1.56	1.52		Mg	1.56	1.75
	Al	80.7	87.12		Al	49.75	62.18
